# Compassionate Use of GC5131 (Hyperimmunoglobulin) Therapy in Critically Ill Patients Diagnosed with COVID-19: A Case Series and Review of Literature

**DOI:** 10.3390/v13091826

**Published:** 2021-09-14

**Authors:** Sunha Choi, Soyoon Hwang, Kitae Kwon

**Affiliations:** 1 Division of Pulmonary Disease, School of Medicine, Kyungpook National University, Kyungpook National University Chilgok Hospital, Daegu 47404, Korea; sunha20@gmail.com; 2 Division of Infectious Diseases, School of Medicine, Kyungpook National University, Kyungpook National University Chilgok Hospital, Daegu 47404, Korea; sangod86@naver.com

**Keywords:** COVID-19, immunoglobulin, convalescence, plasma, clinical effectiveness

## Abstract

Presently, the use of convalescent plasma and hyperimmunoglobulin obtained from individuals who have recovered from coronavirus disease 2019 (COVID-19) has proved to potentially provide passive antibody-based immunity, thereby leading to several clinical trials to develop an immune-based COVID-19 treatment. However, the therapeutic efficacy of hyperimmunoglobulin in critically ill patients with COVID-19 remains unknown. On 23 October 2020, we first administered GC5131 in a compassionate-use program to critically ill patients at the Kyungpook National University, Chilgok Hospital, Korea. Since then, five more critically ill patients were treated with GC5131 in this compassionate-use program in our hospital up until 17 December 2020. We retrospectively reviewed the clinical responses of six critically ill patients diagnosed with COVID-19 who received the hyperimmunoglobulin concentrate, GC5131, which was produced by the Green Cross Corporation. After the administration of GC5131, five patients died due to an exacerbation of COVID-19 pneumonia. GC5131 was ineffective when administered to critically ill patients with COVID-19. Nevertheless, we propose that to expect a therapeutic effect from GC5131, it should be administered as early as possible to avoid the excessive inflammatory response phase in patients with severe and advanced COVID-19 infection. This step was difficult to achieve in the real world due to the time required for decision making and the process of the compassionate-use program.

## 1. Introduction

It has been hypothesized that the primary cause of the worsening condition of severely ill patients with coronavirus disease 2019 (COVID-19) is a cytokine storm. Dysregulated release of inflammatory products is a feature of this disease, leading to organ failure and acute respiratory distress syndrome [1]. Hence, clinical trials on immune-based medications that suppress hyperinflammation are being conducted for their use as a treatment for COVID-19 [2]. Among these medications, administering convalescent plasma (CP), which is obtained by extracting blood plasma from donors who have recovered from COVID-19, has been shown to significantly improve the clinical outcomes of hospitalized patients with the disease [3]. CP provides neutralizing antibodies that stop the viral replication process by blocking the binding of receptors; thus, preventing wall fusion, or preventing the uncoating of viruses once inside the cytoplasm [4]. Moreover, CP provides passive immunomodulatory mediators, such as anti-inflammatory cytokines, clotting factors, natural antibodies, and other undefined proteins that allow recipients to control excessive inflammatory cascades induced by these infectious agents [5].

Both CP and hyperimmunoglobulin therapy are produced from blood plasma. However, highly purified and concentrated specific antibodies are obtained from a number of individuals for hyperimmunoglobulin therapy, which results in high titers of these specific antibodies which are used against a microorganism [6]. As such, hyperimmunoglobulin has a standard and high-titer of antibody, but with less volume when compared with CP. Moreover, the hyperimmunoglobulin substance does not include complement proteins, plasma factors, procoagulants, or antifibrinolytics [6]. Furthermore, although some problems exist with the production protocol of using CP, such as a lack of guidelines, lack of donors, and difficulties in donor scheduling [7], hyperimmunoglobulin therapy can easily be used because it is a formulation already produced by a pharmaceutical company. In this case, GC5131 is a type of hyperimmunoglobulin concentrate produced by the Green Cross Corporation (Yongin-si, Gyeonggi-do, Korea). The Korean Ministry of Food and Drug Safety has individually authorized the compassionate use of GC5131 as an unlicensed, investigational therapeutic agent for critically ill patients with COVID-19 when no other treatments are available [8].

On 23 October 2020, we administered GC5131 as part of the first compassionate-use program in Korea to a critically ill patient who did not respond to other treatments, including dexamethasone and remdesivir. Since then, five more critically ill patients have been treated with GC5131 at our hospital up until 17 December 2020. Few reports exist on the therapeutic efficacy of hyperimmunoglobulin therapy in critically ill patients with COVID-19. Therefore, herein, we report on six cases of critically ill patients with COVID-19 who received GC5131 as part of a compassionate-use program.

## 2. Materials and Methods

This study was conducted at the Kyungpook National University, Chilgok Hospital, a public tertiary hospital in Daegu, Korea. Between 23 October 2020, and 17 December 2020, six patients diagnosed with COVID-19 were administered GC5131, after approval was obtained for the compassionate-use program of GC5131. In the approved cases, the treatment consisted of a single dose of 10,000 mg of GC5131 in a 250 mL solution. The infusion was started at 0.01–0.02 mL/kg/min for 30 min, after which it was gradually increased to 0.06 for the remainder of the infusion.

The electronic medical records of each patient were also reviewed and analyzed retrospectively. We obtained demographic information, clinical symptoms, and laboratory results, including the management and treatment outcome data of each patient. Moreover, we used the recorded cycle threshold (Ct) value of each patient to analyze the trend of decreasing viral load before and after the administration of GC5131. Subsequently, Ct values from the patients’ nasopharyngeal and sputum specimens were also measured using real-time reverse transcription–PCR (RT–PCR), targeting the RNA-dependent RNA polymerase (RdRp) gene. RNA was extracted from clinical samples with an automated nucleic acid extraction platform Libex (Xian Tianlong Science & Technology, Xi’an, China). Severe acute respiratory syndrome coronavirus 2 (SARS-CoV-2) was detected by RT–PCR using a PowerChekTM 2019 nCoV Real-Time PCR Kit (KogeneBiotech, Seoul, Korea) and a CFX96 Real-Time PCR detection system (Bio-Rad, Berkeley, CA, USA).

The institutional review board approved this case series as a retrospective cohort study, which used data collected from routine clinical practice and waived the requirement to obtain any informed consent.

## 3. Results

Table 1 shows the clinical characteristics of the COVID-19 patients who received GC5131. Of the six critically ill patients diagnosed with COVID-19, four were male and two were female. The median age was 69.5 years (range: 45.0–78.0 years). Furthermore, the median time from symptom onset to diagnosis was 5 days (range: 1–8 days), whereas the median time from diagnosis to GC5131 administration was 9 days (range: 4–31 days). Moreover, the median time from application to administration of the GC5131 compassionate-use program was 5.5 days (range: 2–9 days), and the median time from symptom onset to administration was 14 days (range: 9–37 days).

**Figure 1:**
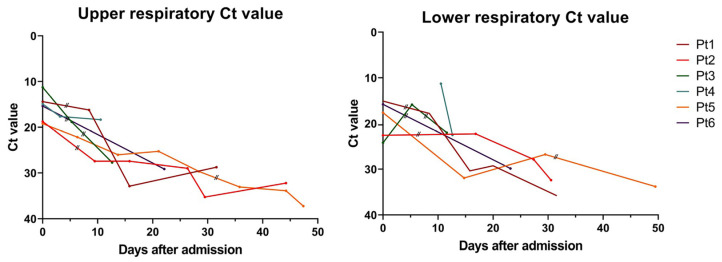
The cycle threshold (Ct) values of SARS-CoV2 detected in patients’ upper (**left**, nasopharyngeal) and lower respiratory specimens (**right**, expectorate sputum), who underwent hyperimmunoglobulin (GC5131) therapy. A double slash represents the day each patient received the therapy. Patient 4 received the therapy on the 14th day, so the graph does not show the date of medication.

After the administration of GC5131, all six patients required invasive mechanical ventilation, but one patient refused this treatment and was treated with a high-flow nasal cannula instead. None of the patients experienced adverse events associated with GC5131, which can include rashes, increased liver enzyme levels, or convulsions. However, we were unable to check for subjective symptoms such as nausea or headache in four of the patients because they were unconscious. Subsequently, one patient recovered and was discharged 16 days after GC5131 administration, whereas the other five patients died. The proximate cause of death in all five patients who died was respiratory failure due to an exacerbation of COVID-19 pneumonia. The surviving patient was clinically improving even before the GC5131 therapy. Hence, we were unable to determine the effectiveness of GC5131 in critically ill patients with COVID-19. Furthermore, no significant change in the trend of decreasing viral load before and after the administration of GC5131 was observed (Figure 1).

## 4. Discussion

The effectiveness of CP and hyperimmunoglobulin in patients diagnosed with COVID-19 is proposed to differ according to the time of transfusion, illness severity, volume administered, neutralizing antibody concentration, risk of antibody-dependent enhancement, and other adverse events [9]. In particular, it is considered that the concentration of the neutralizing antibodies, which are expected to block a step of the viral replication cycle by binding to the surface of viral particles, thereby reducing their infectivity, determines the efficacy of CP and hyperimmunoglobulin therapies for patients with COVID-19 [10,11]. However, a recent study found that CP administration with high titers of neutralizing antibodies does not benefit patients who were hospitalized for COVID-19 [12]. It also found no significant difference in the optical density ratio of total immunoglobulin (Ig), IgM SARS-CoV-2 receptor-binding domain antibodies, and viral neutralization capacities between baseline levels of hospitalized patients who had been symptomatic for a median of 10 days, and levels in donors at week 6 post infection [12]. Considering current data, the seroconversion rate and the antibody levels increase rapidly during the first 2 weeks [13], and active therapeutic effects of humoral anti-SARS-CoV-2 responses are most expected before 10 days after the onset of symptoms, which are proposed to decrease rapidly after that [12,14,15].

Therefore, to expect active therapeutic effects of GC5131, it should be administered as soon as possible after the onset of symptoms. In our cases, the period from symptom onset, diagnosis, compassionate-use decision, and approval processes for the administration of GC5131 was long in the early cases and then gradually shortened. Nevertheless, only one patient received the GC5131 therapy within 10 days after the onset of symptoms (Table 1). Moreover, as faster and higher neutralizing antibody responses occurred in severe cases [12,16,17], it was difficult to observe the effect of GC5131 administration in the critically ill cases. To address this problem, the approval process for the compassionate use of GC-5131, including obtaining documentation for patient consent and submission of patient medical certificates, has been simplified and shortened. As a result, the time it takes to receive approval has been shortened (Table 1).

A systematic review and exploratory meta-analysis of 32 studies, published in 2015, on severe acute respiratory infections (SARIs) including severe acute respiratory syndrome coronavirus (SARS-CoV) and severe influenza, demonstrated the efficacy of CP and hyperimmunoglobulin on mortality and reduction in viral load in patients with SARI [18]. Likewise, for COVID-19 infections, a recent propensity score-matched control study showed that the recipients of CP who were not mechanically ventilated at the time of transfusion were significantly less prone to death than their matched controls [19]. However, only a few reports have been presented on whether hyperimmunoglobulin therapy is effective in treating patients with COVID-19. Compared with CP, hyperimmunoglobulin has the advantages of possessing higher titers of the neutralizing antibody, but with reduced volume. Hence, it would be difficult to show its therapeutic effects because it contains few complement proteins and antimicrobial peptides, namely antithrombotic and coagulation factors that influence immunomodulatory effects [6]. Furthermore, although it is unknown which neutralizing antibody or immunomodulatory effect is more important for treating COVID-19, several studies showed that a subgroup of severe COVID-19 patients experienced uncontrolled, excessive inflammatory responses that caused cytokine storm syndromes [14,20]. Studies have also shown that bronchoalveolar lavage of patients with the severe disease contained highly inflammatory monocyte–derived macrophage populations, which were not present in patients with mild disease [21]. Considering these results, it is proposed that anti-inflammatory and immunomodulatory effects critically influence treatments for patients in the advanced stage of severe COVID-19, as viral replication has already decreased, and the disease process is primarily driven by responses from the host, thereby leading to immune dysregulation and immunopathology. As such, hyperimmunoglobulin has little effect on immune response modulating effects, such as blocking proinflammatory cytokines, suppressing inflammatory cytokines, or enhancing anti-inflammatory cytokines. Therefore, hyperimmunoglobulin therapy in our cases is proposed to be not effective in critically ill patients with COVID-19. In addition, in our cases the median time from symptom onset to death in the five patients who died was 23 days (Interquartile range [IQR] 20.5–48.5), which is not significantly different from the 21 days reported for 101 patients who died at an ICU in a hospital in China at the beginning of the COVID-19 pandemic [22]. It may be difficult to construe significant meaning here due to the small number of patients in our case, but hyperimmunoglobulin does not seem to extend the survival period. Currently, Green Cross Pharmacy has stopped commercializing GC5131 and has withdrawn its conditional application for GC5131, since the Korean Ministry of Food and Drug Safety rejected the application for conditional approval of GC5131 for use in plasma therapy for COVID-19 [23].

Recently, in June 2021, the first report evaluating the efficacy of using hyperimmunoglobulin in severe and critical COVID-19 patients was published [24]. In this study, although doses of hyperimmunoglobulin administered to each patient were lower than those used in our cases, which ranged from 0.15 to 0.3 g/kg, it was found that hyperimmunoglobulin therapy improved the chances of survival and reduced the risk of disease progression in 40 severely or critically ill patients with COVID-19 [24]. In that study, the mean days from the onset of symptoms to the administration of hyperimmunoglobulin was 8.37 days, which is earlier than in our cases. Moreover, only one of the 40 patients received invasive mechanical ventilation at the start of treatment, so the condition of patients was less severe compared with those in our study. Hence, the effectiveness of the hyperimmunoglobulin therapy in that study could be observed.

Until recently, many conflicting opinions about the therapeutic effects of CP and hyperimmunoglobulin use in COVID-19 patients have been reported. However, several clinical trials are still ongoing, including participants with varying COVID-19 severities and who are receiving different interventions. Additionally, recently, a type of novel immune-based drug known as neutralizing monoclonal antibodies, designed using biotechnology to avidly bind to the receptor-binding domain of the SARS-CoV-2 spike glycoprotein, is being developed [25]. Another report states that using monoclonal antibody therapies for high-risk ambulatory COVID-19 patients within 7 days of symptom onset was effective in preventing the need for emergency department visits and hospital admissions [26]. Therefore, as the mechanism of monoclonal antibody therapy is also based on blocking and neutralizing the SARS-CoV-2 virus in infected patients, it is not expected to be effective for patients when administered late after the onset of symptoms or for those already in severe or critical conditions. Remdesivir, a nucleoside analogue, which was shown to demonstrate potent inhibition of SARS-CoV-2 replication in vitro, has also not been shown to be effective in terms of improving the overall mortality of critically ill patients with COVID-19 [27,28]. Considering these points, all antiviral agents used against SARS-CoV2 infection are most effective when applied at an early stage of infection, which can interfere with multiple rounds of viral replication [28].

Our study, however, has limitations in interpreting the results because it is a retrospective case series conducted at a single center using a small sample size. Nevertheless, this study is valuable because no other case report or article exists on critically ill COVID-19 patients receiving GC5131.

## 5. Conclusions

In conclusion, GC5131 was not found to be effective in critically ill patients with COVID-19. To expect a therapeutic effect from GC5131, it should be administered as early as possible before the infection becomes critical. However, this step was difficult to achieve in the real world due to the time required for decision-making and the process of the compassionate-use program. Hence, further clinical studies are warranted to determine whether early administration of hyperimmunoglobulin therapy can be helpful in patients with severe COVID-19. Moreover, when considering the administration of antibody-based immunotherapies, including hyperimmunoglobulin, CP, and monoclonal antibody therapies to COVID-19 patients, it is recommended to administer them as early as possible to cause a therapeutic effect.

## Figures and Tables

**Table 1:** Clinical characteristics of patients diagnosed with COVID-19 who underwent the GC5131 therapy.

	Case 1	Case 2	Case 3	Case 4	Case 5	Case 6
Age	78	78	71	68	45	68
Sex	Male	Female	Male	Male	Male	Female
Smoking history	Ex-smoker	No	Ex-smoker	No	No	No
Presenting symptoms	Fever, cough, myalgia	Fever, cough, dyspnea, diarrhea, sore throat	Fever, chills, general weakness	Dyspnea	Fever, diarrhea, general weakness	Cough, dyspnea
Days from symptoms onset to diagnosis	6	1	5	5	8	3
Date the GC5131 was administered	2020.10.23	2020.11.18	2020.11.18	2020.12.12	2020.12.16	2020.12.17
Days from application to administration of GC5131	9	6	6	5	2	3
Days from application to approval for the compassionate use of GC5131	5	5	5	4	1	2
Days from diagnosis to administration of GC5131	31	20	10	8	4	6
Days from symptom onset to administration of GC5131	37	21	15	13	12	9
Severity on admission day	Moderate	Severe	Severe	Severe	Critical	Severe
Severity on day of administration of GC5131	Critical	Critical	Critical	Critical	Critical	Critical
Comorbidities	HTN Old stroke HCMP Asthma Dyslipidemia	HTN Dyslipidemia	Old TB COPD BPH Dyslipidemia	BPH	HTN Colon cancer	Obesity
HbA1c	5.7	6.5	6.5	6.1	7.7	6.4
Treatment (Before) ^a^						
Antivirals	Remdesivir	Remdesivir	None	Remdesivir	None	Remdesivir
						
Antibiotics or antifungal agents	Ceftriaxone, piperacillin-tazobactam, zithromax, meropenem, teicoplanin, doripenem, levofloxacin, fluconazole, colistin	Meropenem, moxifloxacin, piperacillin-tazobactam, teicoplanin, metronidazole, amikacin, fluconazole	Ceftriaxone, zithromax	Piperacillin-tazobactam, zithromax	Meropenem	Ceftriaxone, zithromax, meropenem, vancomycin
						
Steroid	Dexam	Dexam	Dexam	Dexam	Dexam	Dexam
						
Oxygen delivery devices	Ventilator	HFNC	Ventilator	Ventilator	Ventilator	Ventilator
Treatment (After) ^b^						
Antivirals	None	None	None	None	None	None
						
Antibiotics or antifungal agents	Meropenem, doripenem, colistin	Piperacillin-tazobactam, amikacin, teicoplanin, fluconazole	Piperacillin-tazobactam, zithromax, meropenem, vancomycin	Meropenem, levofloxacin, vancomycin, piperacillin-tazobactam, gentamicin, colistin, fluconazole	Meropenem, vancomycin	Meropenem, vancomycin, trimethoprim/sulfamethoxazole
						
Steroid	None	Dexam	Dexam	Dexam	Dexam	Dexam
						
Oxygen delivery devices	Ventilator	HFNC	Ventilator	Ventilator	Ventilator	Ventilator, ECMO
Outcome	Recovery, discharge 20 days after GC5131 administration	Death, 2 days after GC5131 administration	Death, 6 days after GC5131 administration	Death, 45 days after GC5131 administration	Death, 8 days after GC5131 administration	Death, 30 days after GC5131 administration
Cause of death		Respiratory failure	Respiratory failure	Respiratory failure	Respiratory failure	Respiratory failure, catheter related sepsis

BPH, benign prostatic hyperplasia; COPD, chronic obstructive pulmonary disease; CRRT, continuous renal replacement therapy; Dexam, dexamethasone; Dx, diagnosis; ECMO, extracorporeal membrane oxygenation; HCMP, hypertrophic cardiomyopathy; HFNC, high-flow nasal cannula; HTN, hypertension; Old TB, old tuberculosis; Sx, symptom; Tx, treatment. ^a^ Treatments used before GC5131 administration. ^b^ Treatments used after GC5131 administration.

## Data Availability

All data is contained within the article.
